# Chloroplast genomes: diversity, evolution, and applications in genetic engineering

**DOI:** 10.1186/s13059-016-1004-2

**Published:** 2016-06-23

**Authors:** Henry Daniell, Choun-Sea Lin, Ming Yu, Wan-Jung Chang

**Affiliations:** Department of Biochemistry, School of Dental Medicine, University of Pennsylvania, South 40th St, Philadelphia, PA 19104-6030 USA; Agricultural Biotechnology Research Center, Academia Sinica, Taipei, Taiwan

## Abstract

Chloroplasts play a crucial role in sustaining life on earth. The availability of over 800 sequenced chloroplast genomes from a variety of land plants has enhanced our understanding of chloroplast biology, intracellular gene transfer, conservation, diversity, and the genetic basis by which chloroplast transgenes can be engineered to enhance plant agronomic traits or to produce high-value agricultural or biomedical products. In this review, we discuss the impact of chloroplast genome sequences on understanding the origins of economically important cultivated species and changes that have taken place during domestication. We also discuss the potential biotechnological applications of chloroplast genomes.

**Electronic supplementary material:**

The online version of this article (doi:10.1186/s13059-016-1004-2) contains supplementary material, which is available to authorized users.

## Introduction

Chloroplasts are active metabolic centers that sustain life on earth by converting solar energy to carbohydrates through the process of photosynthesis and oxygen release. Although photosynthesis is often recognized as the key function of plastids, they also play vital roles in other aspects of plant physiology and development, including the synthesis of amino acids, nucleotides, fatty acids, phytohormones, vitamins and a plethora of metabolites, and the assimilation of sulfur and nitrogen. Metabolites that are synthesized in chloroplasts are important for plant interactions with their environment (responses to heat, drought, salt, light, and so on) and their defense against invading pathogens. So, chloroplasts serve as metabolic centers in cellular reactions to signals and respond via retrograde signaling [1, 2]. The chloroplast genome encodes many key proteins that are involved in photosynthesis and other metabolic processes.

The advent of high-throughput sequencing technologies has facilitated rapid progress in the field of chloroplast genetics and genomics. Since the first chloroplast genome, from tobacco (*Nicotiana tabacum*), was sequenced in 1986 [3], over 800 complete chloroplast genome sequences have been made available in the National Center for Biotechnology Information (NCBI) organelle genome database, including 300 from crop and tree genomes. Insights gained from complete chloroplast genome sequences have enhanced our understanding of plant biology and diversity; chloroplast genomes have made significant contributions to phylogenetic studies of several plant families and to resolving evolutionary relationships within phylogenetic clades. In addition, chloroplast genome sequences have revealed considerable variation within and between plant species in terms of both sequence and structural variation. This information has been especially valuable for our understanding of the climatic adaptation of economically important crops, facilitating the breeding of closely related species and the identification and conservation of valuable traits [4, 5]. Improved understanding of variation among chloroplast genomes has also allowed the identification of specific examples of chloroplast gene transfer to plant nuclear or mitochondrial genomes, which has shed new light on the relationship between these three genomes in plants.

In addition to improving our understanding of plant biology and evolution, chloroplast genomics research has important translational applications, such as conferring protection against biotic or abiotic stress and the development of vaccines and biopharmaceuticals in edible crop plants. Indeed, the first commercial-scale production of a human blood protein in a Current Good Manufacturing Processes (cGMP) facility was published recently [6]. The lack of conservation of intergenic spacer regions, even among chloroplast genomes of closely related plant species, and the species specificity of regulatory sequences have facilitated the development of highly efficient transformation vectors for the integration and expression of foreign genes in chloroplasts. Because the published literature is rarely cross-referenced, this review highlights the impact of chloroplast genomes on various biotechnology applications. In addition to our enhanced understanding of chloroplast biology, we discuss in depth the roles of chloroplast genome sequences in improving our understanding of intracellular gene transfer, conservation, diversity, and the genetic basis by which chloroplast transgenes are engineered to enhance plant agronomic traits or to produce high-value agricultural or biomedical products. In addition, we discuss the impact of chloroplast genome sequences on increasing our understanding of the origins of economically important cultivated species and changes that occurred during domestication.

## Advances in chloroplast genome sequencing technology

One of the important factors in the rapid advancement of the chloroplast genomics field is improvement in sequencing technologies. In studies conducted before the availability of high-throughput methods, isolated chloroplasts were used for the amplification of the entire chloroplast genome by rolling circle amplification [7–12]. An alternative strategy is to screen bacterial artificial chromosome (BAC) or fosmid libraries using chloroplast genome sequences as probes [13–20]; however, these methods are subject to many challenges, including difficulty in constructing good-quality BAC or fosmid libraries, large numbers of PCR reactions, and the possibility of contamination from other organellar DNA [21–32]. The PCR approach is also difficult to apply to species that have no relatives whose chloroplast genomes have been sequenced or those with highly rearranged chloroplast genomes.

The development of next-generation sequencing (NGS) methods provided scientists with faster and cheaper methods to sequence chloroplast genomes. Moore and colleagues [33] first reported using NGS to determine chloroplast genome sequences, in *Nandina* and *Platanus*. Although multiple NGS platforms are available for chloroplast genome sequencing [34], Illumina is currently the major NGS platform used for chloroplast genomes [21, 32, 35, 36] because it allows the use of rolling circle amplification products [35, 37]. Investigators can then use bioinformatics platforms to perform de novo assembly without the need for reference genome sequences; from these assemblies it is possible to identify consensus chloroplast genome sequences [32]. A third-generation sequencer, the PacBio system which uses single molecule real-time (SMRT) sequencing, is now widely used in chloroplast genome sequencing [38–43]. Its advantage is long read lengths [44], which facilitate de novo genome assembly, particularly in the four chloroplast junctions between the inverted repeat (IR) and single-copy regions.

The low accuracy (~85 % of the raw data) of the long reads produced by the PacBio platform [45] can be corrected by combining the latest chemistry with a hierarchical genome assembly process algorithm; accuracy rates as high as 99.999 % can be achieved after such post-error corrections [46]. Accuracy can also be increased using Illumina short reads [42]. In a study of *Potentilla micrantha*, sequencing with the Illumina platform produced seven contigs covering only 90.59 % of the chloroplast genome; by contrast, using the PacBio platform with error correction, the entire genome was successfully assembled in a single contig [39].

## Chloroplast genome structure

**Fig. 1:**
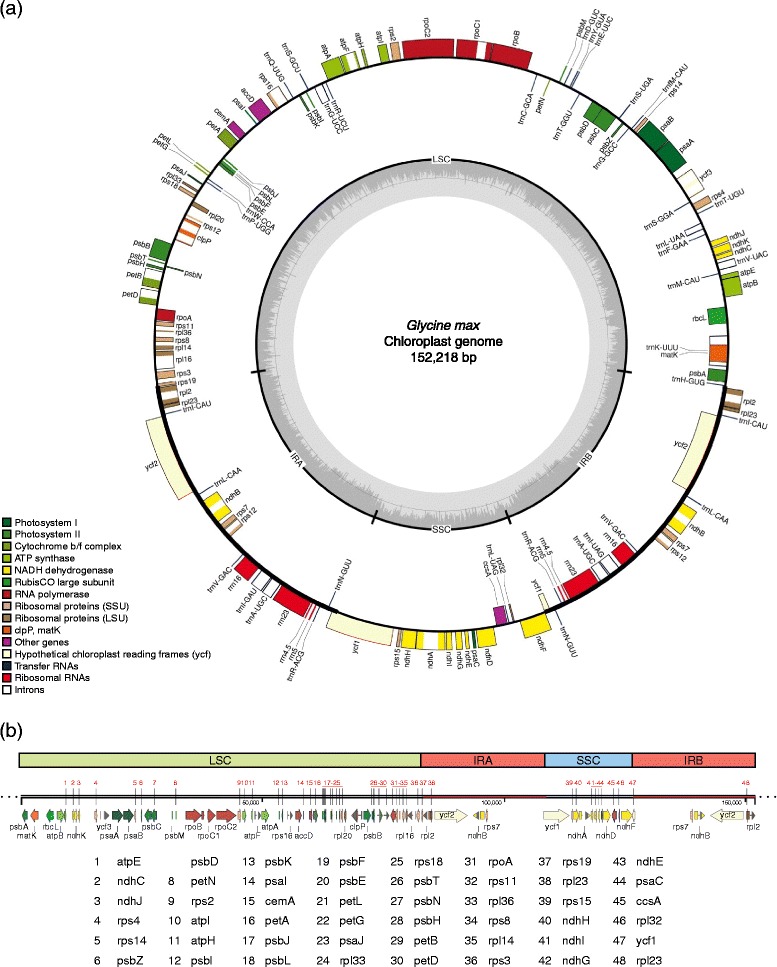
Map of the soybean (*Glycine max*) chloroplast genome. This genome was used to engineer biotic stress tolerance against insects and herbicides. The quadripartite structure includes two copies of an IR region (*IRA* and *IRB*) that separate large single-copy (*LSC*) and small single-copy (*SSC*) regions [18]. **a** Circular form. The GC content graph (*gray circle* inside) marks the 50 % threshold of GC content. **b** Linear form. Different *colors* indicate genes in different functional groups. *IR* inverted repeat, *LSU* large subunit, *SSU* small subunit

The chloroplast genomes of land plants have highly conserved structures and organization of content; they comprise a single circular molecule with a quadripartite structure that includes two copies of an IR region that separate large and small single-copy (LSC and SSC) regions (Fig. 1a, b). The chloroplast genome includes 120–130 genes, primarily participating in photosynthesis, transcription, and translation. Recent studies have identified considerable diversity within non-coding intergenic spacer regions, which often include important regulatory sequences [13]. Despite the overall conservation in structure, chloroplast genome size varies between species, ranging from 107 kb (*Cathaya argyrophylla*) to 218 kb (*Pelargonium*), and is independent of nuclear genome size (Table 1). Certain lineages of land-plant chloroplast genomes also show significant structural rearrangements, with evidence of the loss of IR regions or entire gene families. Furthermore, there is also evidence for the existence of linear chloroplast genomes, as illustrated in Fig. 1b. The percentage of each form within the cell varies in different reports [47, 48].

**Table 1:** Alphabetical list of crop and tree species that have complete annotated chloroplast genome sequences

Species	Common name	Accession	Genome size (bp)	Uses	Reference(s)
Crops					
*Acorus gramineus*	Sweet flag	NC_026299	152849	Ornamental	[169]
*Agrostis stolonifera*	Creeping bent grass	NC_008591	136584	Forage	[8]
*Allium cepa*	Onion	NC_024813	153538	Vegetable	[170]
*Ananas comosus*	Pineapple	NC_026220	159636	Fruit	[171]
*Anthriscus cerefolium*	Chervil	NC_015113	154719	Medicinal	[172]
*Artemisia frigida*	Fringed sagewort	NC_020607	151076	Medicinal	[173]
*Atropa belladonna*	Belladonna	NC_004561	156687	Medicinal	[174]
*Brassica napus* (2)*	Canola	NC_016734	152860	Oil	[175]
*Calanthe triplicata*	Black orchid	NC_024544	158759	Flower	[176]
*Cannabis sativa* (2)	Marijuana	NC_027223	153854	Fiber	[177]
*Capsicum annuum* (2)	Pepper	NC_018552	156781	Vegetable	[178]
*Carica papaya*	Papaya	NC_010323	160100	Fruit	[179]
*Catharanthus roseus*	Madagascar periwinkle	NC_021423	154950	Flower	[180]
*Cenchrus americanus*	Pearl millet	NC_024171	140718	Cereals	[181]
*Cicer arietinum*	Chickpea	NC_011163	125319	Vegetable	[7]
*Coix lacryma-jobi*	Job's tears	NC_013273	140745	Cereals	[29]
*Colocasia esculenta*	Taro	NC_016753	162424	Vegetable	[182]
*Cucumis sativus* (3)	Cucumber	NC_007144	155293	Vegetable	[183]
*Curcuma roscoeana*	Jewel of Burma	NC_022928	159512	Medicinal	[184]
*Cymbidium tortisepalum* (5)	Cymbidium orchid	NC_021431	155627	Flower	[55]
*Cypripedium formosanum* (3)	Formosa's lady's slipper	NC_026772	178131	Flower	[32]
*Daucus carota*	Carrot	NC_008325	155911	Vegetable	[9]
*Dendrobium catenatum*	Dendrobium orchid	NC_024019	152221	Flower	[56]
*Dieffenbachia seguine*	Dumbcane	NC_027272	163699	Ornamental	[185]
*Digitaria exilis*	White fonio	NC_024176	140908	Cereals	[181]
*Echinochloa oryzicola*	Late barnyard grass	NC_024643	139891	Cereals	[186]
*Ephedra equisetina*	Ma Huang	NC_011954	109518	Medicinal	[187]
*Erycina pusilla*	Mini orchid	NC_018114	143164	Flower	[36]
*Fagopyrum esculentum* (2)	Common buckwheat	NC_010776	159599	Cereals	[188]
*Festuca arundinacea* (4)	Kentucky fescue	NC_011713	136048	Forage	[189]
*Fragaria vesca* (6)	Wild strawberry	NC_015206	155691	Fruit	[190]
*Glycine max* (9)	Soybean	NC_007942	152218	Oil	[18]
*Glycyrrhiza glabra*	Common liquorice	NC_024038	127943	Medicinal	[74]
*Gossypium barbadense* (22)	Sea island cotton	NC_008641	160317	Fiber	[69]
*Guizotia abyssinica*	Ramtilla	NC_010601	151762	Bird seed	[191]
*Helianthus annuus* (9)	Common sunflower	NC_007977	151104	Oil	[192]
*Heliconia collinsiana*	Platanillo	NC_020362	161907	Ornamental	[193]
*Hordeum vulgare*	Barley	NC_008590	136462	Cereals	[8]
*Hyoscyamus niger*	Henbane	NC_024261	155720	Medicinal	[194]
*Ipomoea batatas*	Sweet potato	NC_026703	161303	Vegetable	[195]
*Ipomoea purpurea*	Common morning glory	NC_009808	162046	Ornamental	[196]
*Lactuca sativa*	Lettuce	NC_007578	152765	Vegetable	[197]
*Lilium superbum*	Turk's-cap lily	NC_026787	152069	Flower	[198]
*Lolium multiflorum* (2)	Ryegrass	NC_019651	135175	Lawn	[199]
*Lotus japonicus*	Birdsfoot trefoil	NC_002694	150519	Forage	[200]
*Manihot esculenta*	Cassava	EU117376	161453	Starch crop	[20]
*Masdevallia picturata* (2)	Masdevallia orchid	NC_026777	157423	Flower	[32]
*Musa textilis*	Banana	NC_022926	161347	Fruit	[184]
*Nicotiana tabacum* (4)	Tobacco	Z00044	155943	Tobacco	[3]
*Nuphar advena*	Spatterdock	NC_008788	160866	Medicinal	[201]
*Nymphaea alba* (2)	White water-lily	NC_006050	159930	Flower	[24]
*Oncidium* hybrid	Oncidium	NC_014056	146484	Flower	[54]
*Oryza sativa* (6)	Rice	X15901	134525	Cereals	[202]
*Panax ginseng* (2)	Ginseng	NC_006290	156318	Medicinal	[203]
*Panicum virgatum*	Switchgrass	NC_015990	139619	Biofuel	[204]
*Paphiopedilum armeniacum* (2)	Slipper orchid	NC_026779	162682	Flower	[32]
*Parthenium argentatum*	Guayule	NC_013553	152803	Biofuel	[205]
*Pelargonium* (2)	Geranium	NC_008454	217942	Flower	[206]
*Phalaenopsis* hybrid (3)	Phalaenopsis orchid	NC_007499	148964	Flower	[51]
*Phaseolus vulgaris*	Kidney bean	NC_009259	150285	Bean	[78]
*Pisum sativum*	Pea	NC_014057	122169	Vegetable	[76]
*Raphanus sativus*	Radish	NC_024469	153368	Vegetable	[207]
*Ravenala madagascariensis*	Traveller's tree	NC_022927	166170	Ornamental	[184]
*Ricinus communis*	Castor bean	NC_016736	163161	Oil	[208]
*Saccharum* hybrid (2)	Sugarcane	NC_005878	141182	Sugar	[209]
*Salvia miltiorrhiza*	Redroot sage	NC_020431	151328	Medicinal	[210]
*Secale cereale*	Rye	NC_021761	114843	Cereals	[64]
*Sesamum indicum*	Sesame	NC_016433	153324	Oil	[211]
*Solanum lycopersicum* (11)	Tomato	NC_007898	155461	Vegetable	[13]
*Solanum tuberosum*	Potato	DQ231562	155312	Starch crop	[212]
*Sorghum bicolor* (2)	Sorghum	NC_008602	140754	Cereals	[8]
*Spinacia oleracea*	Spinach	NC_002202	150725	Vegetable	[213]
*Trifolium grandiflorum* (8)	Large-flower hop clover	NC_024034	125628	Forage	[74]
*Triticum aestivum* (6)	Bread wheat	NC_002762	134545	Cereals	[63]
*Vanilla planifolia*	Vanilla	NC_026778	148011	Fruit	[32]
*Vigna radiata* (3)	Mung bean	NC_013843	151271	Bean	[79]
*Zea mays*	Maize	NC_001666	140384	Cereals	[62]
*Zingiber spectabile*	True ginger	NC_020363	155890	Ornamental	[193]
Trees and perennial plants					
*Abies koreana*	Fir	NC_026892	121373	Wood	[214]
*Actinidia chinensis* (2)	Kiwifriut	NC_026690	156346	Fruit	[215]
*Amentotaxus formosana*	Taiwan catkin yew	NC_024945	136430	Timber	[216]
*Araucaria heterophylla*	Norfolk island araucaria	NC_026450	146723	Timber	[217]
*Bambusa multiplex* (4)	Golden goddess bamboo	NC_024668	139394	Ornamental	[91]
*Bambusa oldhamii*	Green bamboo	NC_012927	139350	Vegetable	[28]
*Berberis bealei*	Beale's mahonia	NC_022457	164792	Ornamental	[218]
*Bismarckia nobilis*	Bismarck palm	NC_020366	158210	Ornamental	[193]
*Buxus microphylla*	Japanese box	NC_009599	159010	Ornamental	[219]
*Calocedrus formosana*	Taiwan incense-cedar	NC_023121	127311	Timber	[220]
*Calycanthus floridus*	Carolina-allspice	NC_004993	153337	Medicinal	[23]
*Camellia oleifera* (13)	Tea oil plant	NC_023084	156971	Oil	[221]
*Camellia reticulata*	To-tsubaki	NC_024663	156971	Flower	[222]
*Carludovica palmata*	Toquilla palm	NC_026786	158545	Fiber	[198]
*Castanea mollissima*	Chestnut	NC_014674	160799	Fruit	[14]
*Cathaya argyrophylla*	Cathaya	NC_014589	107122	Timber	[223]
*Cedrus deodara*	Cedar	NC_014575	119299	Timber	[223]
*Cephalotaxus wilsoniana* (2)	Wilson plum yew	NC_016063	136196	Timber	[224]
*Chrysobalanus icaco*	Coco plum	NC_024061	162775	Fruit	[225]
*Citrus sinensis* (2)	Orange	NC_008334	160129	Fruit	[12]
*Cocos nucifera*	Coconut	NC_022417	154731	Oil	[226]
*Coffea arabica*	Coffee	NC_008535	155189	Beverage	[10]
*Corymbia gummifera* (4)	Red bloodwood	NC_022407	160713	Timber	[227]
*Corynocarpus laevigata*	Karaka nut	NC_014807	159202	Fruit	[37]
*Cryptomeria japonica*	Sugi	NC_010548	131810	Timber	[228]
*Dendrocalamus latiflorus*	Sweet giant bamboo	NC_013088	139394	Vegetable	[28]
*Elaeis guineensis*	African oil palm	NC_017602	156973	Oil	[229]
*Eucalyptus globulus* (32)	Eucalyptus	NC_008115	160286	Timber	[230]
*Hevea brasiliensis*	Rubber tree	NC_015308	161191	Rubber	[231]
*Jasminum nudiflorum*	Winter jasmine	NC_008407	165121	Ornamental	[232]
*Jatropha curcas*	Barbados nut	NC_012224	163856	Biofuel	[233]
*Juniperus bermudiana* (4)	Bermuda juniper	NC_024021	127659	Timber	[234]
*Larix decidua*	European larch	NC_016058	122474	Timber	[224]
*Licania sprucei* (3)	Licania	NC_024065	162228	Ornamental	[225]
*Liquidambar formosana*	Chinese sweetgum	NC_023092	160410	Timber	[30]
*Liriodendron tulipifera*	Tulip tree	NC_008326	159886	Timber	[235]
*Metasequoia glyptostroboides*	Dawn redwood	NC_027423	131887	Timber	[236]
*Millettia pinnata*	Indian beech	NC_016708	152968	Ornamental	[81]
*Morus indica* (3)	White mulberry	NC_008359	158484	White mulberry	[237]
*Nageia nagi*	Asian bayberry	NC_023120	133722	Timber	[220]
*Nandina domestica*	Heavenly bamboo	NC_008336	156599	Ornamental	[33]
*Nerium oleander*	Oleander	NC_025656	154903	Ornamental	[238]
*Olea europaea* (5)	Olive	NC_015604	155862	Oil	[239]
*Phoenix dactylifera*	Date palm	NC_013991	158462	Fruit	[240]
*Phyllostachys edulis* (4)	Moso bamboo	NC_015817	139679	Timber	[89]
*Picea sitchensis* (3)	Sitka spruce	NC_011152	120176	Timber	[35]
*Pinus taiwanensis* (12)	Taiwan red pine	NC_027415	119741	Timber	[241]
*Platanus occidentalis*	American sycamore	NC_008335	161791	Ornamental	[33]
*Podocarpus lambertii* (3)	Podocarpus	NC_023805	133734	Ornamental	[242]
*Populus alba*	White poplar	NC_008235	156505	Timber	[243]
*Prinsepia utilis*	Himalayan cherry	NC_021455	156328	Ornamental	[244]
*Prunus persica* (6)	Peach	NC_014697	157790	Fruit	[14]
*Pseudophoenix vinifera*	Florida cherry palm	NC_020364	157829	Ornamental	[193]
*Pseudotsuga sinensis*	Chinese douglas	NC_016064	122513	Timber	[224]
*Pyrus pyrifolia* (2)	Chinese pear	NC_015996	159922	Fruit	[245]
*Quercus rubra* (4)	Oak	NC_020152	161304	Timber	[246]
*Sapindus mukorossi*	Soapberries	NC_025554	160481	Medicinal	[247]
*Taiwania cryptomerioides* (2)	Taiwania	NC_016065	132588	Timber	[224]
*Theobroma cacao*	Cacao tree	HQ336404	160604	Beverage	[14]
*Vaccinium macrocarpon*	Large cranberry	NC_019616	176045	Fruit	[248]
*Vitis vinifera*	Wine grape	NC_007957	160928	Fruit	[19]
*Wollemia nobilis*	Wollemia	NC_027235	145630	Timber	[249]

*The number of species in the same genus as the listed species that have sequenced and annotated chloroplast genomes is shown in parentheses

Like the genes, the introns in land-plant chloroplast genomes are generally conserved, but the loss of introns within protein-coding genes has been reported in several plant species [49], including barley (*Hordeum vulgare*) [8], bamboo (*Bambusa* sp.) [28], cassava (*Manihot esculenta*) [20], and chickpea (*Cicer arietinum*) [7]. The proteins encoded by genes in which intron loss is known to occur have diverse functions; they include an ATP synthase (atpF), a Clp protease (clpP), an RNA polymerase (rpoC2), and ribosomal proteins (rpl2, rps12, and rps16) [49]. The majority of reported intron losses have been observed in specific plant groups or species, although some examples of intron loss (such as that in *clpP*) occur in diverse plant species, including monocots (Poaceae), eudicots (Onagraceae and Oleaceae) and gymnosperms (*Pinus*) [49].

## Diversity of chloroplast genome sequences

At higher taxonomic levels (family level), protein-coding regions and conserved sequences of the chloroplast genome can be used for phylogenetic analysis and domestication studies [49]. Earlier phylogenetic analyses utilized partial chloroplast DNA sequences. The use of variable regions or multiple DNA fragments dramatically enhanced the utility of these analyses but there is insufficient information in these sequences to provide the high-resolution necessary to differentiate closely related taxa, particularly some within-species taxa whose taxonomic relationships are unclear. Complete chloroplast genome sequences are valuable for deciphering phylogenetic relationships between closely related taxa and for improving our understanding of the evolution of plant species.

In this section, we discuss several examples of comparisons of chloroplast genomes, within and between crop species, that have provided unique insight into evolutionary relationships among taxa. We also discuss the origin and geographic distribution of economically important species, as well as their adaptations to different climatic conditions and the use of genome information in their breeding and conservation.

A key application of the chloroplast genome in agriculture is the identification of commercial cultivars and the determination of their purity. DNA barcodes derived from the chloroplast genome can be used to identify varieties and in the conservation of breeding resources. Success in breeding is determined by genetic compatibility and chloroplast genomes serve as a valuable tool for identifying plants that are likely to be closely related and, therefore, genetically compatible. Understanding the genetic relationships between cultivated crops and their wild relatives informs efforts to introduce specific advantageous traits into cultivated crops. In the section below, we discuss how chloroplast genomes have been used to elucidate the evolutionary relationships and domestication history of a few major crops and how this informs breeding programs.

### Breeding

The Orchidaceae is a large family that encompasses about 6–11 % of all angiosperms [50] and is important in floriculture. Many commercially important orchid species belong to the subfamily Epidendroideae and chloroplast genomes of several species from this subfamily have been sequenced [51–58]. Because it is easy to perform inter-generic crossing in orchids and because the record of breeding is sometimes incomplete, it is often difficult to validate the parental origin of commercially important varieties [54]. Corrected parental information is important for breeding and variety identification. In an investigation of the Oncidiinae, a subtribe within the Epidendroideae, PCR products derived from eight conserved regions in 15 commercial varieties resolved their phylogenetic relationship at the species level [54] and helped to resolve putative errors in parental origin. Parental records had indicated that *Odontoglossum* ‘Violetta von Holm’, *Odontoglossum* ‘Margarete Holm’ and *Odontocidium* ‘Golden Gate’ are derived from the same female parent (*Odontoglossum bictoniense*) but phylogenetic analyses of ‘Violetta von Holm’ did not correlate with those of ‘Golden Gate’ or ‘Margarete Holm’ [54]. A possible reason for inconsistencies between the chloroplast DNA-based phylogenetic tree and the parental record is chloroplast capture. Chloroplast capture is the introgression of chloroplasts from one species into another after intrageneric and intergeneric hybridization [59]. Although chloroplast genomes provide useful information for phylogenetic analyses involving closely related taxa, chloroplast capture by hybridization may distort phylogenetic relationships if captured chloroplast genomes or genes included therein are used [60]. The use of both nuclear and chloroplast genomes can provide more complete phylogenies [4, 61].

### Phylogenetic studies

There are several published chloroplast genomes from cereals, including those from sorghum (*Sorghum bicolor*), barley [8], maize (*Zea mays*) [62], wheat (*Triticum aestivum*) [63], rye (*Secale cereale*) [64], and rice (*Oryza sativa*) [65]. Rice is one of the world's most important crops and is the primary carbohydrate source for the global human population (http://www.ers.usda.gov/topics/crops/rice.aspx). The *Oryza* species are classified into ten genome types, including six diploids (AA, BB, CC, EE, FF, and GG) and four allotetraploids (BBCC, CCDD, HHJJ, and HHKK). Attempts to clarify the evolutionary relationships between cultivated rice and its wild relatives remain contentious and inconclusive [4]. For example, there are two wild species that have an AA genome in Australia, *Oryza meridionalis* (annual) and *Oryza rufipogon* (perennial). *Oryza sativa* was domesticated from Asian *O. rufipogon* 10,000 years ago [65]. Nevertheless, analysis of complete Australian and Asian wild rice chloroplast genomes indicated that Australian *O. rufipogon* chloroplast genomes are more similar to those of Australian *O. meridionalis* than to those of Asian *O. rufipogon* [65–67]. Using 19 chloroplast genomes of *Oryza* AA genome species, a robust phylogenetic tree was established, which will aid in improving rice crops and in conservation strategies [4, 5].

Cotton is the most important textile fiber crop and the first cotton (*Gossypium hirsutum*) chloroplast genome was published in 2006 [11]. The diploid *Gossypium* species comprise eight genome groups (A to G and K genomes). *Gossypium hirsutum* (upland cotton), the most widely planted cotton species in the world, is an allotetraploid of the ancestral A and D genome species [68]. Chloroplast genome sequences are available for 22 *Gossypium* species and these can be used to glean information about the evolution and domestication of this crop [11, 68, 69] (Table 1). Simple sequence repeat primers were used to investigate 41 species of *Gossypium*, including all eight genome groups and allotetraploid species [70]. The results indicated that two modern A-genome species, *Gossypium herbaceum* and *Gossypium arboretum*, were not cytoplasmic donors of tetraploid (AD) species; instead, the AD genome species originated from an extinct ancestor species of the modern A genome [68, 70].

### Domestication

Information on chloroplast genomes is useful for understanding the domestication of several crops, particularly legumes [71]. The chloroplast genome structure of legumes is very interesting; it contains multiple rearrangements, including large inverted segments and loss of inverted repeats [72]. An example is a 51-kb inversion that was first identified in the soybean (*Glycine max*) chloroplast genome sequence [18] before being reported in most members of the subfamily Papilionoideae [7, 73–77]. A 78-kb reversion was subsequently confirmed in *Phaseolus* and *Vigna* chloroplast genomes [78, 79]. More recently, 36-kb [80] and 5.6-kb [81] inversions inside the 51-kb inversion were identified. There are many important genes within these inverted regions but no gene is disturbed and plant survival and performance are not affected. These unique characteristics are not only very useful in phylogenetic studies [82] but also provide important information for chloroplast transformation in legumes. Chloroplast structure is also important for the design of primers needed in the amplification of sequences for further domestication and phylogenetic analysis.

*Citrus* is one of the most commercially important fruit genera. In 2006, the first *Citrus* chloroplast genome, that of sweet orange (*Citrus × sinensis*), was published [12] and this served as a reference genome for subsequent publications [83, 84]. Phylogenetic analysis of 34 chloroplast genomes of *Citrus* (28) and *Citrus*-related genera (6) indicated that citrus fruits have the same common ancestor [84, 85]. In four genes (*matK*, *ndhF*, *ycf1*, and *ccsA*), single-nucleotide variations and insertion/deletion frequencies were clearly higher than average and showed that these genes have been positively selected. The *matK* gene encodes a maturase that is involved in splicing type II introns and the *matK* sequence is often used in phylogenetic and evolutionary studies [84]. Positive selection of *matK* is observed not only in citrus but is common in several other plant species. In fact, more than 30 plant groups have been shown to undergo positive selection of *matK* genes, indicating that the gene is subject to a number of different ecological selective pressures [86]. The *ndhF* gene encodes a subunit of the chloroplast NAD(P)H dehydrogenase (NDH) complex. Chloroplast NDH monomers are sensitive to high light stress, suggesting that the *ndh* genes may also be involved in stress acclimation [87]. These studies indicated that *matK* and *ndhF* show positive selection in Australian species, potentially contributing to their adaptation to a hot, dry climate [84, 85].

Bamboo is an economically and ecologically important forest plant in Asia [88]. Bamboo grows quickly and new culms are regenerated from the rhizome after harvesting, making it a sustainable and ecologically and environmentally friendly crop. The first two bamboo chloroplast genomes have been published [28] and many more bamboo chloroplast genomes are now available [88–93]. Bamboo has a long juvenility and it is difficult to obtain flowers for taxonomic studies; consequently the taxonomic relationships of bamboo have proven challenging to unravel on the basis of traditional reproductive organ morphology. Furthermore, the extremely low rate of sequence divergence meant that the taxonomic and phylogenetic relationships of temperate woody bamboos at lower taxonomic levels proved difficult to resolve [88]. These relationships were eventually resolved with high-resolution phylogenetic trees using 25 bamboo chloroplast genomes [93]. In addition to woody bamboos, chloroplast genomes have also been published for herbaceous bamboo [88, 92]. An interesting phenomenon identified in herbaceous bamboo chloroplast genomes is that of gene transfer from the mitochondrial genome to the chloroplast genome. This was an unusual observation, as the chloroplast genome is thought to be nearly immune to the transfer of DNA from nuclear and mitochondrial genomes [88, 92, 94]. A possible reason for this recalcitrance to DNA transfer is the lack of an efficient DNA uptake system [94]. Prior to its observation in herbaceous bamboo, this phenomenon was only observed in two eudicot chloroplast genomes [94] and in monocots [88, 92].

## Transfer of chloroplast genes to nuclear or mitochondrial genomes

There are three distinct genomes in plant cells: nuclear, mitochondrial, and plastid. Mitochondria are believed to have evolved from a single endosymbiotic event by the uptake of a proteobacterium, whereas chloroplasts evolved from endosymbiosis of a cyanobacterium, after which there was a massive transfer of genes from the chloroplast to the nucleus [95]. There are distinct translation systems in these organelles: nuclear-encoded genes are translated in the cytosol and the protein products are then transported to the locations in which they function, including chloroplasts [96], whereas chloroplast-encoded proteins are directly synthesized within the chloroplast. Multi-subunit functional protein complexes that are involved in photosynthesis or protein synthesis are also assembled within chloroplasts.

Gene content, number, and structure are conserved in the chloroplast genome sequences of most autotrophic land plants [97, 98] but some protein-encoding genes are absent in specific species [49]. The loss of genes such as *infA*, *rpl22*, and *ndh* from the chloroplast genome and their intracellular transfer to the nuclear or mitochondrial genomes provide valuable information for phylogenetic analyses and evolutionary studies. It is very easy to identify the chloroplast origin of genes in plant mitochondrial or nuclear genomes [99, 100] by intracellular gene transfer [32], but this could also lead to erroneous phylogenic relationships when short sequences are used instead of complete chloroplast genome sequences.

**Fig. 2:**
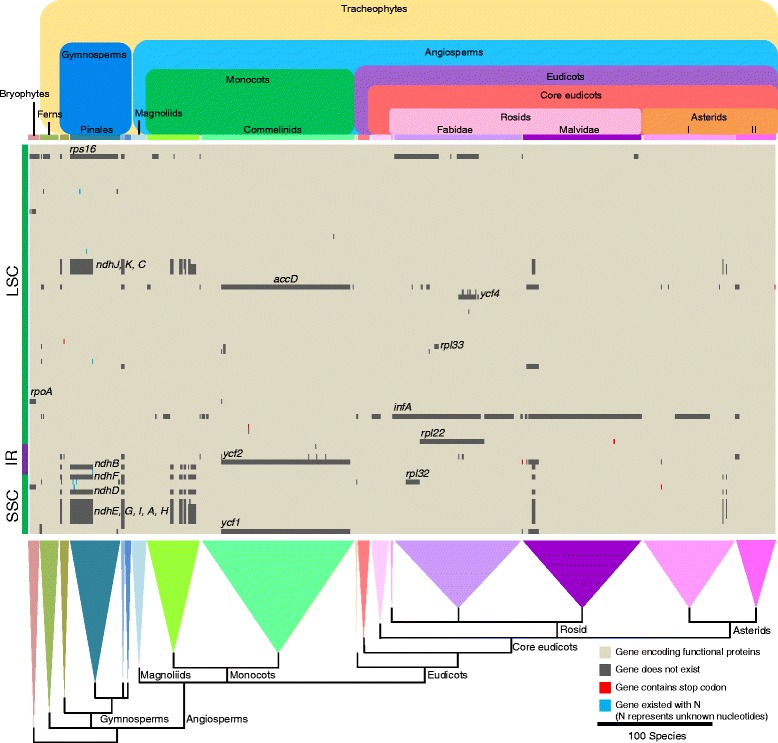
Chloroplast genome structure and gene expression across tracheophytes. These 658 chloroplast genomes were downloaded from NCBI Organelle Resources. The *X-axis* indicates the taxonomy of the chloroplast genome species following the Angiosperm Phylogeny Group III system and NCBI taxonomy. The *bar width* represents 100 species. The *Y-axis* shows the chloroplast genes, which were classified by different chloroplast regions. *Gray boxes* indicate absence of genes. *Red boxes* indicate stop codons in genes. *Blue boxes* indicate unknown nucleotides (*N*) in genes. *IR* inverted repeat, *LSC* large single-copy region, *SSC* small single-copy region

The chloroplast *translation initiation factor 1* (*infA*) is a homolog of the essential gene *infA* in *Escherichia coli* [101, 102]. This gene initiates translation in collaboration with two nuclear-encoded initiation factors to mediate interactions between mRNA, ribosomes, and initiator tRNA-Met [102]. Many parallel losses of chloroplast-encoded *infA* have occurred during angiosperm evolution [102] (Fig. 2). Nuclear-encoded *infA* genes have been identified in *Arabidopsis thaliana*, soybean, tomato (*Solanum lycopersicum*), and ice plant (*Mesembryanthemum crystallinum*) [102]. Protein sequences of nuclear-encoded *infA* in these four species contain chloroplast transit peptides. Studies using soybean and *A. thaliana* infA-GFP proteins have shown that nuclear-encoded *infA* genes are translated in the cytosol and transported into chloroplasts [102]. Many more chloroplast-encoded *infA* deletions have been identified recently (Fig. 2).

There are 57 chloroplast genomes in 26 genera in which the essential gene *rpl22* is reported to have been deleted from the chloroplast and transferred to the nuclear genome (Fig. 2) [14, 103]. Nuclear-encoded *rpl22* contains a transit peptide that is predicted to deliver this protein from the cytosol to chloroplasts. These peptides are diverse, suggesting that there were two independent *rpl22* transfers in the Fabaceae and the Fagaceae [14]. Similar transfer to the nucleus has also been observed for *rpl32* deletion from chloroplast genomes [104–106].

Eleven chloroplast genes encode *ndh* subunits, which are involved in photosynthesis. The ndh proteins assemble into the photosystem I complex to mediate cyclic electron transport in chloroplasts [107, 108] and facilitate chlororespiration [109]. Some autotrophic plants lack functional *ndh* genes in their chloroplast genomes [36, 51, 54, 55, 110–115] (Fig. 2). Unlike the single gene losses described previously, the entire family of *ndh* genes has been deleted in these plants. Seven orchid chloroplast genomes indicated at least three independent *ndh* deletions [32]. Some orchid *ndh* DNA fragments were identified in the mitochondrial genome but the complete *ndh* genes required to translate putative functional protein complexes are absent [32]. In the nuclear genome of Norway spruce, only non-functional plastid *ndh* gene fragments are present [116]. Normal photosynthesis is observed in these *ndh*-deleted species [32, 117]. Furthermore, *ndh*-deleted transformants are autotrophic and produce carbohydrates through photosynthesis [107, 118–121].

Many more chloroplast-gene deletions have been observed, including deletions of *accD*, *ycf1*, *ycf2*, *ycf4*, *psaI*, *rpoA*, *rpl20*, *rpl23*, *rpl33*, and *rps16*; many unique gene deletions have been identified in only one or a few species (*psbJ*, *rps2*, *rps14*, and *rps19*) (Fig. 2). The functions of these genes, phenotypes of their knock-out mutants, and evidence for their transfer are summarized in Additional file 1. Most essential genes that have been lost from chloroplast genomes have been transferred to the nucleus to maintain the plant's photosynthetic capacity, with the exception of *ycf1* and *ycf2*.

In summary, chloroplast genome sequences are most valuable for understanding plant evolution and phylogeny. Databases of not only plant genomes but also plant transcriptomes will be useful in investigating deletion events or the transfer of chloroplast genes to other organellar genomes to complement such deletions.

## Advances in chloroplast genome engineering

In the past century, desirable agronomic traits, including yield enhancement and resistance to pathogens or abiotic stress, were achieved by breeding cultivated crops with their wild relatives. As explained above, chloroplast genome sequences are very useful in the identification of closely related, breeding-compatible plant species. With the advent of modern biotechnology, desirable traits from unrelated species can now be readily introduced into commercial cultivars. Such genetically modified crops have revolutionized agriculture in the past two decades, dramatically reducing the use of chemical pesticides and herbicides while enhancing yield. For most commercial cultivars, herbicide- or insect-resistance genes are introduced into the nuclear genome. There are, however, a few limitations for nuclear transgenic plants, including low levels of expression (<1 % total soluble protein (TSP)) and potential escape of transgenes via pollen.

Engineering the introduction of foreign genes into the chloroplast genome addresses both of these concerns. Just two copies of transgenes are typically introduced into the nuclear genome, whereas up to 10,000 transgene copies have been engineered into the chloroplast genome of each plant cell, resulting in extremely high levels of foreign gene expression (>70 % TSP) [122]. Most importantly, chloroplast genomes are maternally inherited in most cultivated crops, minimizing or eliminating transgene escape via pollen [123].

**Fig. 3:**
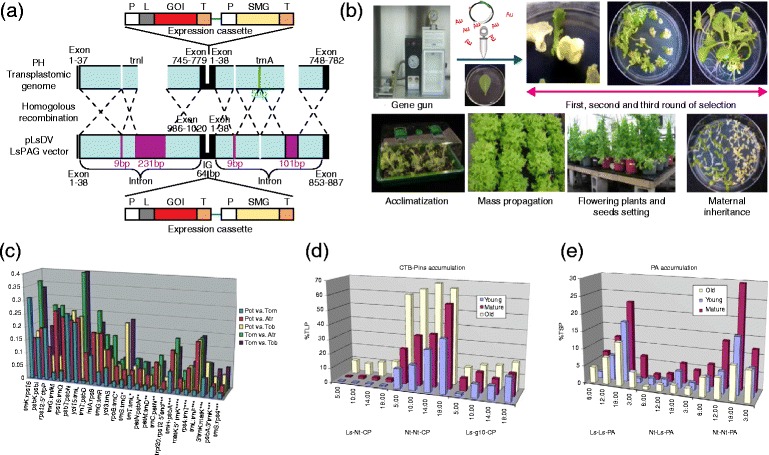
Basic process of chloroplast genetic engineering, diversity in intergenic spacer regions, and impact of transgene integration (endogenous versus heterologous genome sequences). **a** Complexity of heterologous sequence integration into intergenic spacer regions between lettuce and tobacco. The schematic diagram represents recombination between the tobacco transplastomic genome and the lettuce transformation vector [128]. *Purple bars* represent unique lettuce intron sequence; the *green bar* represents unique tobacco intron sequence; *black bars* are exon regions; *blue regions* are looped out sequence. The expression cassette comprises: promoters (*P*), leader sequence (*L*), gene of interest (*GOI*), terminators (*T*), and selectable marker gene (*SMG*). *IG* intergenic spacer region. **b** Basic process of chloroplast genetic engineering. Gene delivery is performed by bombardment with gold microparticles coated with chloroplast vectors, followed by three rounds of selection to achieve homoplasmy. After confirmation of transgene integration, plants are grown in the greenhouse to increase biomass. Chloroplast transgenes are maternally inherited without Mendelian segregation of introduced traits. **c** Comparison of 21 of the most variable intergenic spacer regions among Solanaceae chloroplast genomes. *Atr Atropa*, *Pot* potato, *Tob* tobacco, *Tom* tomato. *Tier 1, **tier 2, and ***tier 3 regions reported in the paper by Shaw et al. [250]. Plotted values were converted from percentage identity to sequence divergence on a scale from 0 to 1 as shown on the *Y-axis*; these values demonstrate a wide range of sequence divergence in different regions. Nucleotide sequences were determined by a bridging shotgun method and genome annotation was performed using the Dual Organellar GenoMe Annotator [13]. **d**, **e** Decrease in the expression of transgenes regulated by heterologous psbA promoters and untranslated regions (UTRs) engineered via tobacco chloroplast genomes. When the lettuce (*La*) *psbA* regulatory region was used in tobacco (*Na*) chloroplasts or vice versa, transgene expression is dramatically reduced. **d** Accumulation of a cholera toxin B subunit (*CTB*) and proinsulin (*Pins*) fusion protein (*CP*) was quantified by densitometry and **e** anthrax protective antigen (*PA*) accumulation was estimated by enzyme-linked immunosorbent assay (ELISA). Total leaf protein (*TLP*) or total soluble protein (*TSP*) data are presented as a function of light exposure and developmental stage. The order of young, mature, and old is different in **d** and **e** because of the accumulation of more CTB-Pins in older leaves and PA in mature leaves [128]. Young (top five), mature (fully grown), and old (bottom three) leaves were fully expanded and were cut from plants grown in the greenhouse for 8–10 weeks

The basic process of chloroplast engineering is explained in Fig. 3a, b. Chloroplast genome engineering is accomplished by integrating foreign genes into intergenic spacer regions without disrupting the native chloroplast genes (Fig. 3a). Two chloroplast genes are used as flanking sequences to facilitate integration of transgene cassettes. Transgene cassettes include a selectable marker gene and gene(s) of interest, both regulated by chloroplast gene promoters and untranslated regions (UTRs; Fig. 3a). Chloroplast genome sequences are essential to build transgene cassettes because they provide both flanking and regulatory sequences. Transgene cassettes that are inserted into bacterial plasmids are called chloroplast vectors and they are bombarded into plant cells using gold particles and a gene gun (Fig. 3b). Because of the presence of chloroplast DNA in the nuclear or mitochondrial genome, transgene cassettes may integrate via homologous or non-homologous recombination events; but any transgenes that are integrated within the nuclear or mitochondrial genome will not be expressed because chloroplast regulatory sequences are not functional in other genomes. If such integration occurs, the transgenes could be easily identified by evaluation of their integration site and eliminated [124].

One of the challenges of creating chloroplast transgenic (transplastomic) plants is the elimination of all untransformed copies (>10,000 per cell) of the native chloroplast genome and replacing them with transformed genomes that contain integrated transgene cassettes. The absence of the native chloroplast genome and the presence of only the modified genomes is referred to as the homoplasmic state, which is typically achieved after two or three rounds of selection (Fig. 3b). The most effective selectable marker used is the *aadA* gene, which confers resistance to streptomycin and spectinomycin. These antibiotics bind specifically to chloroplast ribosomes and disrupt protein synthesis without interfering with any other cellular process. Efforts to transform the chloroplast genome of cereal crops have been mostly unsuccessful. This could be due to the instability of chloroplast DNA in the mature leaves of cereals [47] or to a requirement for better selectable markers [125].

Table 2 provides the first global, comprehensive summary of the power of chloroplast genetic engineering, utilizing valuable information generated by the sequencing of chloroplast genomes described in previous sections. This table includes the most complete list of chloroplast genomes that have been engineered for enhanced agronomic traits or the production of different bio-products, including biopolymers, industrial enzymes, biopharmaceuticals, and vaccines. Within Table 2, transgenes are grouped according to their functions and are organized according to their site of integration. The efficiency of transgene expression is also included in Table 2, providing important information about the regulatory sequences used to express the transgenes.

**Table 2:** Engineering the chloroplast genome for biotechnology applications

Site of integration	Transgenes	Regulatory sequences	Efficiency of expression	Engineered traits or products	Reference(s)
Insect or pathogen tolerance					
trnI/trnA	*Bgl-1*	5′psbA/3′psbA	>160-fold enzyme	Resistance against whitefly and aphid	[141]
trnI/trnA	*Pta*	5′psbA/3′psbA	7.1–9.2 % TSP	Broad-spectrum resistance against aphid, whitefly, Lepidopteran insects, bacterial and viral pathogens	[142]
trnI/trnA	*PelB1*, *PelD2*	5′psbA/3′psbA	~2.42 units mg^−1^ FW	Resistance against *Erwinia* soft rot	[150]
trnI/trnA	*RC1011*, *PG12*	5′psbA/3′	17–38 % TSP	Resistance to *Erwinia* soft rot and tobacco mosaic virus	[140]
trnI/trnA	*cpo*	Prrn/psbA/psbA	NR	Resistance to fungal pathogens in vitro (*Aspergillus flavus*, *Fusarium verticillioides*, and *Verticillium dahliae*) and in planta (*Alternaria alternata*)	[251]
trnI/trnA	*Bt cry2Aa2* operon	Prrn/ggagg/psbA	45.3 % TSP	100 % mortality of cotton bollworm, beet armyworm; cuboidal Bt crystals formation	[137]
trnI/trnA	*Bt cry9Aa2*	Prrn/ggagg/rbcL	~10 % of TSP	Resistance to *Phthorimaea operculella*	[252]
trnI/trnA	*msi-99*	Prrn/ggagg/psbA	21–43 % TSP	Resistance to in planta challenge of *Pseudomonas syringae*, *Aspergillus flavus*, *Fusarium moniliforme*, *Verticillium dahlia*, and *Colletotrichum destructivum*	[253]
trnI/trnA	*sporamin1*, *CeCPI2*, and *chitinase2*	Prrn/TpsbA	0.85–1 % TSP	Resistance against *Spodoptera litura* and *Spodoptera exigua* leaf spot, as well as soft rot diseases	[254]
trnI/trnA	*MSI-99*	Prrn/Trps16	89.75 μg g^−1^ FW	Resistance against rice blast fungus	[255]
trnV/rps12/7	*cry1A(c)*	Prrn/rbcL/rps16	3–5 % of TSP	Resistance to larvae of *Heliothis virescens*, *Helicoverpa zea*, and *Spodoptera exigua*	[256]
trnV/rps12/7	*cry1Ab*	Prrn/T7gene10/rbcL	NR	Resistance to caterpillar of *Anticarsia gemmatalis*	[145]
rbcL/accD	*cry2Aa2*	Prrn/ggagg/psbA	2–3 % of TSP	Resistance to *Heliothis virescens*, *Helicoverpa zea*, and *Spodoptera exigua*	[257]
Abiotic stress tolerance					
trnI/trnA	*tps1*	Prrn/ggagg/psbA	>169-fold transcript	Drought tolerance: growth in 6 % polyethylene glycol and rehydration after 24 days of drought	[258]
trnI/trnA	*merA/merB*	Prrn/ggagg/psbA	NR	Phytoremediation: high level tolerance to the organomercurial compounds, up to 400 μM phenylmercuric acetate	[259]
trnI/trnA	*badh*	Prrn/T7 g10/rps16	93–101 μM g^−1^ FW	Salt tolerance: carrot plants survived up to 400 mM NaCl	[135]
trnI/trnA	*γ-TMT*	Prrn/T7g 10/TpsbA	>7.7 % TSP	Increased salt and heavy metal tolerance, enhanced accumulation of ɑ-tocopherol in seeds	[153]
trnI/trnA	*mt1*	Prrn/T7 g10/Trps16	NR	Phytoremediation: resistant to mercury, up to 20 μm	[260]
trnV/rps12/7	*b-bar1*	Prrn/TrbcL	>7 % TSP	Resistance to the herbicide phosphinothricin	[261]
trnV/rps7/12	*EPSPS*	Prrn/Trps16	>10 % TSP	Resistance to the herbicide glyphosate	[262]
rbcL/accD	*EPSPS/aroA*	Prrn/ggagg/psbA	NR	Resistance to glyphosate (>5 mM)	[129]
rbcL/accD	*mALS*	PpsbA/TpsbA	NR	Tolerant to pyrimidinylcarboxylate, imidazolinon, and sulfonylurea/pyrimidinylcarboxylate herbicides	[263]
rbcL/accD	*Bar*	Prrn/rbcL/psbA	NR	Herbicide resistance: up to 25 μg ml^−1^ glufosinate	[264]
rbcL/rbcL	*Hppd*	psbA/psbA/3′rbcL	5 % TSP	Resistance to herbicide	[265]
rbcL/accD	*panD*	Prrn/rbcL 3′	>4-fold β-alanine	Tolerance to high-temperature stress	[266]
trnfM/trnG	*lycopene β-cyclase*	atpI/rps16	0.28 mg g^−1^ DW	Herbicide resistance and triggers conversion of lycopene	[133]
prs14/trnG	*HTP*, *TCY*, *TMT*	Prrn/T7 g10/TrbcL	NR	Increase in vitamin E in fruit; cold-stress tolerance	[267]
Other agronomic traits					
trnI/trnA	*phaA*	Prrn/psbA/psbA	14.71β-ketothiolase mg^−1^ FW	Engineered cytoplasmic male sterility	[268]
trnI/trnA	*RbcS*	T7g10 or psbA	>150-fold RbcS transcript	Restoration of RuBisCO activity in *rbcS* mutants	[136]
rbcL/accD	*TC*, *γ -TMT*	PpsbA/Trsp16	3 nmol h^−1^ mg^−1^ FW	Vitamin E accumulation in tobacco and lettuce	[269]
rbcL/accD	*CrtZ*, *CrtW*	Prrn/Trps16	NR	Accumulation of astaxanthin fatty acid esters in lettuce	[270]
trnV/orf708	*BicA*	psbA/psbA/psbA	~0.1 % TSP	CO_2_ capture within leaf chloroplasts	[271]
trnV/3′rps12	*Trx f*, *Trx m*	prrn T7G10/rps12	NR	Starch synthesis/chloroplast redox regulation	[272]
trnfM/trnG	*CV-N*	Prrn/T7g10/TatpA	~0.3 % TSP	Increased mRNA stability and protein stability with the expression of CV-N in chloroplasts	[273]
trnI/trnA	*Bgl-1*	5′psbA/3′psbA	44.4 units Bgl1 g^−1^ FW	β-Glucosidase increased enzyme cocktail efficiently to release sugar from paper, citrus peel, and wood	[141]
trnI/trnA	*ubiC*	5′psbA/3′psbA	25 % DW	250-fold higher pHBA polymer accumulation than nuclear transgenic lines	[149]
trnI/trnA	*man 1*	5′psbA/3′psbA	25 units g^−1^ FW	Mannanase increased enzyme cocktail released sugar from paper, citrus peel, and wood	[274]
trnI/trnA	*cutinase* or *swoIlenin*	5′PsbA/3′PsbA	47.7 % reduction of MGDG and DGDG in cutinase and 68.5 % in swollenin	Swollenin enlarged and irreversibly unwound cotton fiber; cutinase showed esterase and lipase activity; used in enzyme cocktails	[275]
trnI/trnA	*bgl1*	5′psbA/3′psbA	14 units mg^−1^ FW	Enzyme cocktails produced glucose from filter paper, pine wood, or citrus peel	[150]
	*swo1*		NR		
	*xyn2*		421 units mg^−1^ FW		
	*Acetyl sylan esterase*		NR		
	*celD*		493 units mg^−1^ FW		
	*celO*		442 units mg^−1^ FW		
	*Lipase*		NR		
	*Cutinase*		15 units mg^−1^ FW		
trnI/trnA	*PMK*, *MVK*, *MDD*, *AACT*, *HMGS*, *HMGRt; IPP*, *FPP*, *ADS*, *CYP71AV1*, *AACPR*	Prrn/PpsbA	0.1 mg g^−1^ FW	Artemisinic acid for several isoprenoid products	[276]
trnI/trnA	*Cel6A*,*Cel6B*	Prrn/rbcL/rbcL	2–4 % TSP	Hydrolyzed crystalline cellulose	[277]
trnfM/trnG	*bgl1C*, *cel6B*, *cel9A*, *xeg74*	Prrn/T7g10/TrbcL	5– 40 % TSP	Cell wall-degrading enzyme activity	[278]
rbcL/accD	*phbC*, *phbA*, *phbB*	Prrn/rbcL 3′	0.16 % DW	Polyhydroxybutyrate (PHB) accumulation in leaves	[279]
rbcL/accD	*crtZ*, *crtW*	Prrn/Trps16	>0.5 % DW	Astaxanthin accumulation	[280]
trnV/rps7	*EGPh*	psbA/psbA/Trps16	25 % TSP	Chloroplast-derived β-1,4-endoglucanase (EGPh) was recovered from dry leaves and digested carboxymethyl cellulose (CMC) substrate	[281]
trnI/trn A	*EX4*	PpsbA/TpsbA	14.3 % TSP	CTB–EX4 showed increased insulin secretion similar to the commercial injectable EX4 in pancreatic β-cells and in mice fed with cells expressing EX4 in chloroplasts	[160]
trnI/trn A	*MBP*	PpsbA/TpsbA	2 % TSP	Amyloid loads were reduced in ex vivo studies in human Alzheimer’s brain and in vivo in Alzheimer’s mice fed with bio-encapsulated CTB–MBP. Abeta was also reduced in retinae and loss of retinal ganglion cells was prevented	[162]
trnI/trn A	*FVIII*	PpsbA/TpsbA	370 mg g^−1^ FW	Feeding of the HC/C2 antigen mixture substantially suppressed T-helper cell responses and inhibitor formation against FVIII in hemophilia A mice	[282]
trnI/trn A	*HSA*	PpsbA/TpsbA	26 % TSP	In vitro chaperone activity of Trx m and Trx f	[283]
trnI/trn A	*EDA*	PpsbA/TpsbA	2.0 % TSP	The vaccine adjuvant EDA from fibronectin retains its proinflammatory properties when expressed in tobacco chloroplasts	[284]
trnI/trn A	*Proinsulin*	PpsbA/TpsbA	47 % TSP in tobacco, 53 % TLP in lettuce	Oral delivery of proinsulin in plant cells lowered glucose levels comparably to injectable commercial insulin	[285]
trnI/trn A	*HSA*	psbA/psbA/psbA	~11 % TSP	First report of human blood protein in chloroplasts; function not evaluated	[286]
trnI/trn A	*IGF*	psbA/psbA/psbA	32.7 % TSP	Promoted growth of cultured HU-3 cells in a dose-dependent manner	[287]
trnI/trnA	*FIX*	PpsbA/TpsbA	1 mg g^−1^ DW (0.56 % TLP)	Oral delivery of CTB-FIX lettuce cells suppressed inhibitor formation against FIX in hemophilia B mice	[6]
trnI/trnA	*FIX*	Ppsba/TpsbA	3.8 % TSP; 0.4 mg g^−1^ FW	Tolerance induction via complex immune regulation, involving tolerogenic dendritic and T-cell subsets	[288]
trnI/trnA	*GAA*	Ppsba/TpsbA	5.7 mg g^−1^ DW	Reduced toxic antibody responses in enzyme replacement therapy in Pompe mice	[289]
trnI/trnA	*ACE2* *Ang-(1–7)*	PpsbA/TpsbA	CTB–ACE2: 2.14 % TLP CTB-Ang1–7: 8.7 % TLP	Oral delivery of ACE2 and Ang (1–7) significantly improved cardiopulmonary structure and functions, decreased the elevated right ventricular systolic blood pressure and improved pulmonary blood flow in animals with induced pulmonary hypertension	[161]
trnI/trn A	*BACE*	Prrn/TpsbA	2.0 % TSP	Immunogenic response against the BACE antigen in mice	[290]
trnI/trn A	*IFNα2b*	Prrn/TpsbA	3 mg g^−1^ FW	Protected cells against VSV CPE and HIV; increased MHC I antibody on splenocytes and total number of natural killer cells and protected mice from a highly metastatic lung tumor	[291]
trnI/trn A	*CTB-pins*	Prrn/T7g10/TpsbA and rps16	16 % TSP in tobacco, 72 % TLP in lettuce	CTB-proinsulin-fed non-obese diabetic mice significantly decreased inflammation (insulitis); insulin-producing β cells in pancreatic islets were highly protected, increased in insulin production with lower blood or urine glucose levels; increased expression of immunosuppressive cytokines	[128, 292]
rbcL/accD	*IFN-γ*	PpsbA/TpsbA	6 % TSP	Protection of human lung carcinoma cells against infection by encephalomyocarditis virus	[293]
rbcL/accD	*hTrx*	PpsbA/Trps16	1 % TSP	Protected mouse from hydrogen peroxide	[294]
rbcL/accD	*A1AT*	PpsbA/TrbcL	2 % TSP	Binds to porcine pancreatic elastase	[295]
rbcL/accD	*TGFβ3*	Prrn/T7g10/psbC	12 % TLP	Inhibits mink lung epithelial cell proliferation	[296]
trnV/3′rps12	*hCT-1*	Prrn/G10L/Trps16	5 % TSP	Biologically active on human hepatocarcinoma cell line	[297]
trnV/rps7/12	*hST*	PpsbA or Prrn/G10L/Trps16	0.2–7.0 % TSP	Promotes growth of Nb2 cells in a dose-dependent manner	[298]
trnfM/trnG	*pal*, *cpl-1*	Prrn/T7g10/TpsbA	~30 % TSP	Bacteriolytic activity and kills *Streptococcus pneumoniae*, the causative agent of pneumonia	[299]
trnI/trn A	*ESAT-6*	5′psbA/3′psbA	~7.5 % TSP	Hemolysis of red blood cells and GM1 binding	[165]
trnI/trn A	*AMA1*	5′psbA/3′psbA	7.3 % TSP in tobacco, 13.2 % TSP in lettuce	Long-term immunity against cholera challenge; inhibition of malarial parasite; protection correlated with IgA and IgG1	[164]
trnI/trn A	*MSP1*	5′psbA/3′psbA	10.1 % TSP in tobacco, 6.1 % TSP in lettuce		
trnI/trn A	*2 L21*	5′psbA/3′psbA	6.0 % TSP		
trnI/trn A	*Pag*	5′psb/3′psbA	~29.6 % TSP	Macrophage lysis assay, systemic immune response, toxin neutralization assay, mice survived (100 %) challenge with lethal doses of anthrax toxin	[300, 301]
trnI/trn A	*L1*	PpsbA/TpsbA	20–26 % TSP	Induced systemic immune response and produced neutralizing antibodies in mice	[302]
trnI/trnA	*RA4*	PpsbA/T psbA	0.2 % TLP	Oral administration elicited both mucosal and systemic Th1/Th2 responses to reduce *Toxoplasma* parasite load	[303]
trnI/trnA	*rFaeG*	PpsbA/TrbcL	>1 % DW	Transplastomic plants expressing the rFaeG protein could possibly be used for delivery of an oral vaccine against porcine F4+ ETEC infections	[304]
trnI/trn A	*F1-V*	Prrn/TpsbA	14.8 % TSP	Orally immunized mice heavily challenged with plague (*Yersinia pestis*) were protected better than those given IP injections	[305]
trnI/trn A	*CTB-2 L21*	PpsbA/TpsbA	31.1 % TSP	Immunogenic in mice following IP or oral administration	[306]
trnI/trnA	VP8*	psbA/psbA/Trps16	600 μg g^−1^ FW	Induced strong immune response and virus neutralization	[307]
trnI/trn A	*CtxB*	Prrn/ggagg/TpsbA	4.1 % TSP	Efficient GM1 ganglioside-binding	[308]
trnI/trn A	*LTB*	Prrn/ggagg/TpsbA	2.5 % TSP	GM1 ganglioside-binding assay	[309]
trnI/trn A	*LecA*	Prrn/T7g10/TpsbA	7 % TSP	Systemic immune response in mice	[310]
trnI/trn A	*BACE*	Prrn/TpsbA	2.0 % TSP	Immunogenic response against the BACE antigen in mice	[290]
rbcL/accD	*OspA*, *OspA-T*	PpsbA/TpsbA	1–10 % TSP	Systemic immune response and protection against *Borrelia burgdorferi* (Lyme disease)	[311]
trnN/trn R	*LTB*	Prrn/T7g10/TrbcL	2.3 % TSP	GM1 ganglioside-binding assay; oral immunization partially protected mice from cholera toxin challenge	[312]
trnN/trnR	*DPT*	Prrn/T7g10/TrbcL	0.8 % TSP	Immunogenic in orally inoculated mice with freeze-dried chloroplast-derived multi-epitope DPT protein	[313]
trnN/trnR	*C4V3*	Prrn/T7g10/TrbcL	~15 μg mg^−1^ DW	Plant-derived C4V3 has elicited both systemic and mucosal antibody responses in mice, as well as CD4+ T cell proliferation responses	[314]
trnN/trnR	*L1*	Prrn/TrbcL	>2 % of TSP	Proper folding and display of conformational epitopes for L1 in the fusion protein by antigen capture ELISA	[315]
trnfM/trnG	*p24*	Prrn/T7g10/TrbcL	~4 % TSP	Induced strong CD4+ and CD8+ T-cell responses in mice	[316]
trnGtrnfM	*HEV E2*	Prrn/psbA/TpsbA	1.09 ng μg^−1^ TSP	Immune response in mice against hepatitis E virus	[317]
trnH/trnK	*CSFV E2*	Prrn/TpsbA	1–2 % TSP	Immune response in mice against swine fever	[318]
rrn16/rps12/7	*TetC*	Prrn/T7 g10/TrbcL atpB/TrbcL	10–25 % TSP	Mice developed systemic immune response and survived the tetanus toxin challenge	[319]
rrn16/trnI	*E7*	PpsbA/Trps	3–8 % TSP	Several therapeutic HPV-specific E7-based vaccine formulations have been tested in animal models and some have advanced into clinical trials	[320]

*Abbreviations*: *Ang (1–7)* Angiotensin (1–7), *BACE* human b-site APP cleaving enzyme, *Bgl* β-glucosidase, *CPE* carbapenemase-producing Enterobacteriaceae, *CTB* cholera toxin B subunit, *DGDG* digalactosyldiacylglycerol, *DPT* diphteria, pertussis, tetanus, *DW* dry weight, *EDA* extra domain A-fibronectin, *ELISA* enzyme-linked immunosorbent assay, *ETEC* enterotoxigenic *Escherichia coli*, *EX4* exendin-4, *FVIII* coagulation factor VIII, *FW* fresh weight, *HPV* human papilloma virus, *IP* intraperitoneal, *MBP* myelin basic protein, *MGDG* monogalactosyldiacylglycerol, *NR* not recorded, *RbcS* small subunit of RuBisCO, *RuBisCO* ribulose-1,5-bisphosphate carboxylase/oxygenase, *TLP* total leaf protein, *TSP* total soluble protein, *VSV* vesicular stomatitis virus

## Impact of sequence diversity in the chloroplast genome on transgene integration

Figure 3a shows examples of transplastomic genomes that have been transformed with either an endogenous or a heterologous flanking sequence. Every single nucleotide change in the heterologous sequence was subsequently edited out and corrected to achieve 100 % homology to the native sequence within the intergenic spacer region (Fig. 3a). The repetitive editing process significantly reduces the efficiency of transgene integration when using heterologous flanking sequences. This challenge is made even more difficult by inadequate conservation of intergenic spacer regions, even within the same family. Figure 3c shows comparisons of 21 of the most variable intergenic spacer regions; only four of the >150 spacer regions, including the trnl/trnA spacer region, are conserved among members of the Solanaceae. Among grass chloroplast genomes, not a single intergenic spacer region is conserved [8]. This necessitates construction of species-specific chloroplast vectors using endogenous flanking sequences and underscores the need to sequence the chloroplast genomes of economically important crop species.

## Ideal sites in the chloroplast genome for transgene integration

The selection of a suitable intergenic spacer region from among more than 100 sites found in each chloroplast genome is a major concern. Statements on the lack of positional effects in the transplastomic literature are common and are used to contrast chloroplast genetic engineering with nuclear transgene integration, which is often associated with profound differences in the expression of transgenes dependent on their site of integration. Evidence shows, however, that there are also positional effects within the chloroplast genome (Table 2). IR regions are found in duplicate in most chloroplast genomes; therefore, transgenes should be inserted within the IR region instead of the SSC or LSC regions because this should double the copy number of transgenes. Integration of a transgene cassette into one copy of the IR facilitates integration into the other copy, thereby enhancing selection pressure to achieve homoplasmy through this copy correction mechanism, a characteristic feature of the chloroplast genome [126–128]. Therefore, the site of integration plays a crucial role in transgene expression level and in enhancing homoplasmy under selection by antibiotics. Most importantly, in all sequenced chloroplast genomes within a single plant species, the DNA sequence in one copy of the IR is identical to that in the other copy, without any exception (Table 1).

An early controversy in the chloroplast genetic engineering field was the suitability of transcriptionally silent spacer regions, where native genes (for example, *rbcL*/*accD*) are located on opposite strands of the chloroplast genome, or transcriptionally active spacer regions, where native genes (for example, *trnA*/*trnI*) are located within operons on the same strand. After a herbicide resistance gene was introduced into the transcriptionally active spacer region for the first time [129], most subsequent studies preferentially used this site of integration (Table 2). The integration of transgenes into the transcriptionally active spacer region (trnl/trnA) has led to 25-fold higher expression of transgenes compared with the transcriptionally silent spacer region (rbcl/accD) [130], possibly due to the presence of multiple promoters (heterologous and endogenous) that enhance transcription. Introns present within *trnI/trnA* genes (used as flanking sequences) also provide efficient processing of native or foreign transcripts. The *trnA* gene intron includes a chloroplast origin of replication and produces more copies of the template (chloroplast vectors) for integration of the transgene cassette [131]. In fact, among 114 transgenes in different plant species in Table 2, 71 are integrated at the *trnA*/*trnI* site of the chloroplast genome, confirming the unique advantages of this site [127, 129, 130].

## Role of chloroplast genome regulatory sequences in transgene expression

In addition to the site of integration, regulatory sequences located upstream (promoter, 5′ UTR) and downstream (3′ UTR) of transgenes play a major role in determining their expression level. The *psbA* regulatory region, first used almost 25 years ago [131], still appears to be the best option for use in an expression cassette, as the *psbA* gene encodes the most highly translated protein in the chloroplast [132] and it can also mediate light-induced activation of translation [128]. Indeed, almost all highly expressed transgenes (>70 % TSP, >25 % dry weight) utilize the *psbA* regulatory region; among 114 transgenes expressed via the chloroplast genome, 84 use the *psbA* regulatory sequence (Table 2). Other endogenous regulatory sequences that are used include *rbcL* and *atpA*, which result in lower transgene expression levels than the *psbA* promoter/5′ UTR.

Using regulatory regions from photosynthetic genes has the advantage of light regulation, making them ideal for transgene expression in photosynthetic organs (leaves; Fig. 3d, e). However, when the lettuce *psbA* regulatory region was used in tobacco chloroplasts or vice versa, transgene expression was dramatically reduced (Fig. 3d, e) [128]. Nucleotide differences within the *psbA* 5′ UTR between tobacco and lettuce (*Lactuca sativa*) resulted in changes that decreased the interaction of RNA-binding proteins and produced variation in the size of the stem, bulge, and terminal loop of the UTR [128]. In addition, most regulatory proteins (including sigma factors that bind to the promoter region) are nuclear encoded and transported to chloroplasts. This underscores a caveat associated with using regulatory sequences for transgene expression: the need to make species-specific chloroplast vectors to accommodate highly specific regulatory region-binding proteins.

**Fig. 4:**
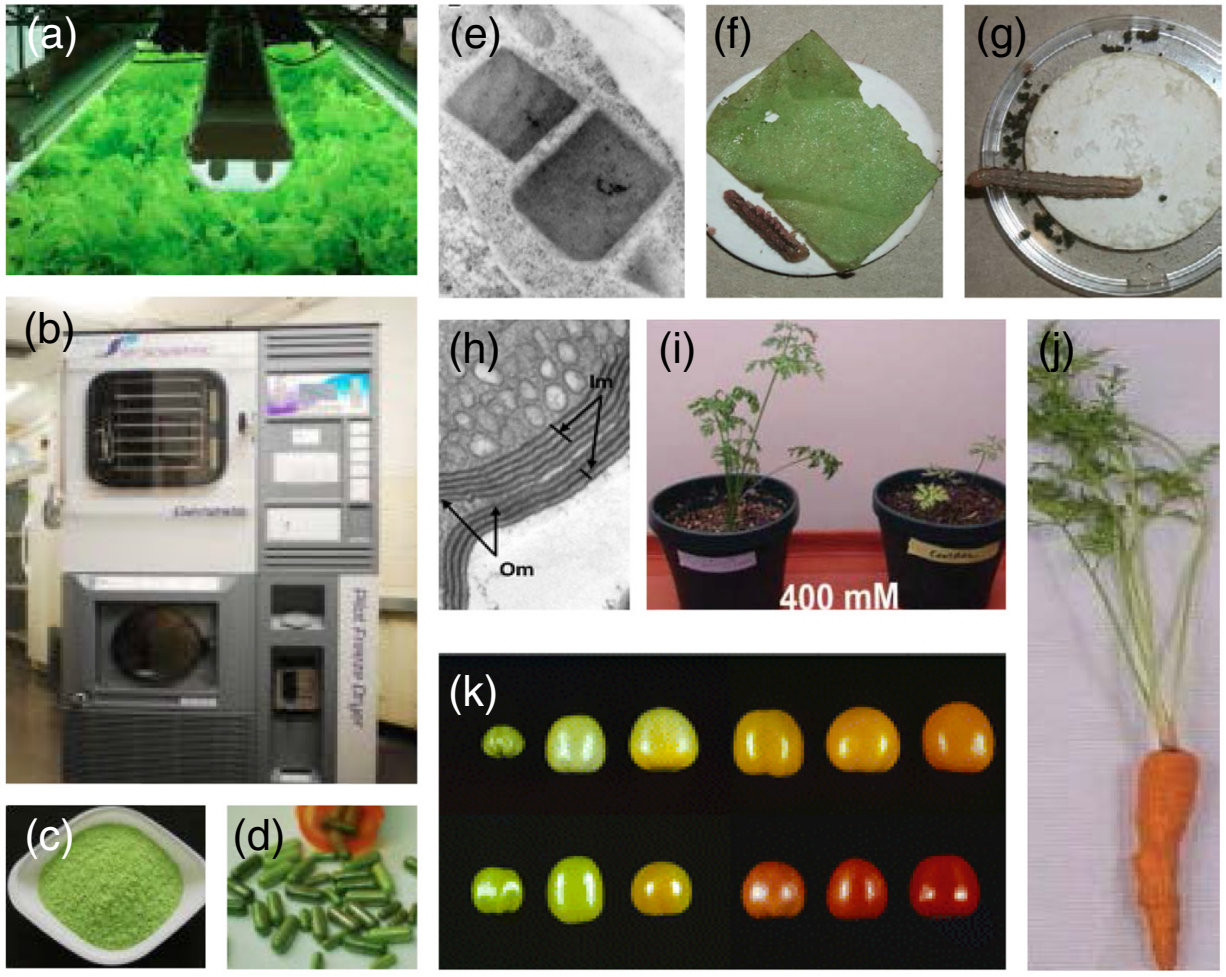
Engineering the chloroplast genome to confer biotic/abiotic stress tolerance or expression of high-value products. **a**–**d** Industrial production of blood clotting factor IX (FIX) bioencapsulated in lettuce plants in a hydroponic cGMP facility. **a** Biomass production of FIX-expressing plants. **b**–**d** Steps in capsule preparation. After harvesting and lyophilization of fresh leaves, freeze-dried FIX-accumulating leaves were powdered and prepared as capsules [6]. **e**–**g** Overexpression of the Bt cry2Aa2 operon in chloroplasts leads to the formation of the Bt insecticidal crystal protein. In bioassays with the *Helicoverpa zea*, **f** eating the transplastomic leaf kills the caterpillar, while **g** the control leaf is consumed by the growing caterpillar [137]. **h** Ultrastructure of the chloroplast envelope membrane of transplastomic γ-tocopherol methyltransferase (γ-TMT) tobacco plants shows the formation of multiple layers of inner envelope membranes as the result of γ-TMT overexpression [153]. **i**, **j** Expression of *BETAINE ALDEHYDE DEHYDROGENASE* (*BADH*) in carrot plants. **i** Transgenic carrot plants thrived in soil irrigated with 400 mM sodium chloride, whereas untransformed carrot plants showed retarded growth in the presence of salt. **j** Carrot roots from transplastomic plants [135]. **k** Phenotypes of tomato fruits from transplastomic tomato plants expressing lycopene β-cyclase transgenes compared with wild-type plants. Fruits were harvested at different ripening stages. Orange color of ripe fruits indicates efficient conversion of red lycopene into orange β-carotene (provitamin A) [154]

Heterologous regulatory sequences are necessary for transgene expression that is independent of cellular control, especially in non-photosynthetic organs such as fruits and edible roots, where chloroplast protein synthesis is poor [133]. A heterologous UTR (T7 *gene10*) was first evaluated for expression in leaves [127, 134] and was subsequently tested in non-green tissues. When the expression of *BETAINE ALDEHYDE DEHYDROGENASE* (*BADH*) was regulated by the T7 *gene10* UTR in carrot (*Daucus carota*) plants, 75 % of the expression level in leaves was observed in non-green edible roots, conferring the highest level of salt tolerance (400 mM NaCl) found in the published literature (Fig. 4i, j) [135]. Although T7 *gene10* has been successfully used to engineer salt tolerance in non-green tissues, its expression level is not as high as that of the *psbA* regulatory sequence in leaves [136]. The only other heterologous UTR that expressed transgenes at high levels is that from the *Bacillus thuringiensis* (*Bt*) operon [137]. Use of this operon produced the highest level of insecticidal toxin protein (52 % TLP) ever reported in the published literature [137]. These high levels of toxin accumulation in chloroplasts could result from the combination of high-level expression and protein stability; the Bt protein formed cuboidal crystals within chloroplasts (Fig. 4e) due to co-expression of a chaperone that facilitates folding. When fed, transplastomic leaves, cotton bollworm (*Helicoverpa* sp.) were killed with a single bite of leaf and insects that had 40,000-fold increased resistance to Bt were also killed (Fig. 4f, g). Nevertheless, expression of this transgene in tomato fruit is very poor [133, 138, 139] and further research is needed to enhance transgene expression in fruits.

## Engineering the chloroplast genomes for biotechnology applications

### Conferring stress tolerance

In the past decade, chloroplast genetic engineering has focused primarily on the overexpression of target genes with the potential to enhance biotic stress tolerance, which is very important for plant protection and yield enhancement. Yield loss due to insect pests can be very serious in many countries. In addition to cotton bollworm resistance conferred by hyper-expression of Bt protein in chloroplasts [137], there are many other striking recent examples of improved biotic stress tolerance. Retrocyclin-101 and Protegrin-1 protect against *Erwinia* soft rot and tobacco mosaic virus (TMV), which result in yield loss in several cultivated crops [140]. Whitefly and aphid resistance has been accomplished by expressing β-glucosidase [141], which releases insecticidal sugar esters from hormone conjugates. Multiple resistances against aphids, whiteflies, lepidopteran insects, and bacterial and viral pathogens were achieved by expressing the *Pinellia ternata agglutinin* (*PTA*) gene in the chloroplast genome [142]. More than 40 transgenes have been stably integrated into and expressed within the chloroplast genome, conferring important agronomic traits, including insect resistance in edible crops cabbage (*Brassica oleracea*) [143], soybean [144, 145], and eggplant (*Solanum melongena*) [146].

More recently, scientists have begun to explore new strategies to downregulate specific target genes. One such approach is to express double-stranded RNAs (dsRNAs) within the chloroplast genome and to use RNA interference (RNAi) to confer the desired agronomic traits, mainly resistance to insects that cause severe yield loss. This strategy has been demonstrated by expressing long or short dsRNAs that activate RNAi and disrupt target genes in insects, providing efficient protection against insects without the need for chemical pesticides. One such example is the suppression of three essential proteins required for insect survival—lepidopteran chitin synthase (Chi), cytochrome P450 monooxygenase (P450), and V-ATPase—using dsRNAs in the tobacco chloroplast system [147]. Each dsRNA was expressed independently in chloroplasts and leaves were fed to insects. The transcription level of target genes in *Helicoverpa* insects decreased to almost undetectable levels in the midgut, resulting in a significant reduction in the net weight of larvae and in pupation rate [147]. Transplastomic potato plants producing *β-actin*-targeting long dsRNA were lethal to Colorado potato beetle (*Leptinotarsa decemlineata*) larvae, providing yet another crop protection mechanism [148].

### Synthesis of enzymes and biomaterials

In addition to improved resistance against both biotic and abiotic stress, the chloroplast genome has been engineered to produce useful enzymes, biomaterials, and biofuels, or even to enhance biomass. The first report of metabolic engineering using chloroplast genomes produced the highest level of the poly(p-hydroxybenzoic acid (pHBA) polymer (25 % dry weight) in normal healthy plants despite the diversion of a major metabolic intermediate [149]. The first use of plant-derived enzyme cocktails for the production of fermentable sugars from lignocellulosic biomass was accomplished recently [150]. Unlike the single biofuel enzymes previously expressed in chloroplasts, nine different genes from bacteria or fungi were expressed in *E. coli* or tobacco chloroplasts using a new technique that enabled the insertion of fungal genes with several introns, eliminating the need to prepare cDNA libraries. Industrial fermentation systems are currently limited by high cost and low production capacity; chloroplast-derived enzyme cocktails offer several striking advantages, including significantly reduced cost, improved stability of chloroplast-derived enzymes, and no need for enzyme purification. Interestingly, expression of β-glucosidase released hormones from conjugates, resulting in elevated phytohormone levels and increased biomass [141], an unexpected outcome of enzyme expression.

### Enhancing nutrition

Seed oils, such as those from soybean, rapeseed (*Brassica napus*), and maize, are the major dietary source of vitamin E. They have very low α-tocopherol content but relatively high levels of γ-tocopherol. Only a few seed oils, such as sunflower (*Helianthus annuus*) seed oil, contain high levels of α-tocopherol, an important precursor of vitamin E [151]. γ-Tocopherol is the biosynthetic precursor of α-tocopherol, suggesting that the α-tocopherol biosynthetic pathway catalyzed by γ-tocopherol methyl transferase (γ-TMT) is the rate-limiting step [152]. Engineering of the *γ-tmt* gene into the chloroplast genome resulted in the formation of multiple layers of the inner chloroplast envelope (Fig. 4h) due to γ-TMT overexpression, with around tenfold higher conversion of γ-tocopherol to α-tocopherol in seeds [153]. Likewise, introducing *lycopene β-cyclase* genes into the tomato plastid genome increased the conversion of lycopene into provitamin A (β-carotene), with obvious phenotypic changes (Fig. 4k) [154].

### Biopharmaceuticals

At present, protein drugs are extremely expensive; for example, >90 % of the global population cannot afford insulin, a drug needed to treat the global diabetes epidemic. The high cost of protein drugs is due to their production in prohibitively expensive fermentation systems (which cost more than $450–700 million to build depending on their capacity [155, 156]), prohibitively expensive purification from host proteins, the need for refrigerated storage and transport, and the short shelf-life of the final product. Protein drugs made by plant chloroplasts overcome most of these challenges because they do not require expensive fermentation systems and are produced in federal drug administration (FDA)-approved hydroponic greenhouses (Fig. 4a) [157]. Lettuce leaves expressing protein drugs are lyophilized and stored indefinitely at ambient temperature without losing their efficacy (Fig. 4b–d) [6]. The plant cell wall protects protein drugs from acids and enzymes in the stomach because human enzymes do not digest plant cell wall glycans. Human gut microbes, however, have evolved to break down every glycosidic bond in the plant cell wall and therefore release the protein drug into the gut lumen, directing its delivery to the blood or immune system [158, 159].

Oral delivery of several human therapeutic proteins expressed in chloroplasts is highly efficacious in the treatment of several human diseases, including diabetes, cardiovascular disease, pulmonary hypertension, and Alzheimer’s disease. Most proteins were expressed in tobacco chloroplasts for initial evaluation and were subsequently expressed in lettuce chloroplasts for advancing them to the clinic. Oral delivery of exendin-4, which modulates the secretion of insulin in a glucose-dependent manner, lowered glucose in diabetic animals by stimulating the production of insulin in a manner similar to that of the injectable drug [160]. Oral delivery of angiotensin-converting enzyme 2 (ACE2) and angiotensin (Ang) (1–7) significantly improved cardiopulmonary structure and function, decreased elevated right ventricular systolic blood pressure, and improved pulmonary blood flow in animals with induced pulmonary hypertension [161]. Oral delivery of plant cells expressing ACE2 and Ang (1–7) also reduced endotoxin-induced uveitis (EIU) and dramatically decreased cellular infiltration and retinal vasculitis, as well as damage and folding in experimental autoimmune uveoretinitis [158]. It is also possible to orally deliver protein drugs across the blood–brain barrier to the Alzheimer’s brain to remove plaques [162].

The first industrial-scale production of human blood clotting factor in a cGMP facility was reported recently [6] (Fig. 4a–d). In a 1000 ft^2^ hydroponic cGMP facility, it is possible to produce up to 30,000 doses for a 20-kg pediatric patient. Clotting factor made in lettuce was stable for up to 2 years when lyophilized cells were stored at ambient temperature, completely eliminating the need for the cold chain. This enables the first commercial development of an oral drug and addresses the extremely expensive purification, cold storage and transportation, and short shelf-life of current protein drugs. Oral delivery of a broad dose range was effective in the prevention of antibody formation after injection of clotting factor IX (FIX), further facilitating human clinical studies.

### Vaccines against infectious diseases

The current iteration of vaccines, using attenuated bacteria or viruses, offer protection against major infectious diseases but they also present major challenges. For example, the oral polio vaccine that is used around the globe has caused severe polio resulting from mutations and recombination with other viruses [163]. In addition, all current vaccines require cold storage and transportation, making distribution in developing countries a major challenge. Many of these challenges can be overcome by using chloroplasts.

One successful chloroplast-derived vaccine conferred dual immunity against cholera and malaria in animal studies [164]. Cholera is a major disease causing high mortality, with the only licensed vaccine being not only expensive but also limited in its duration of protection. No vaccine is currently available for malaria. The cholera toxin-B subunit (CTB) of *Vibrio cholerae* was fused to the malarial vaccine antigen apical membrane antigen-1 (AMA1) and merozoite surface protein-1 (MSP1) and expressed in lettuce or tobacco chloroplasts. While no suitable models exist to test human malaria, a cholera toxin challenge using mice immunized with chloroplast-expressed CTB was highly effective and provided the longest duration of protection in the published literature [164]. These early results show that chloroplasts are ideal for producing low-cost booster vaccines against several infectious diseases [165] for which the global population has been primed previously (Table 2), but lack of an oral priming strategy is still a major limitation in this field.

## Moving forward

It is amazing that the chloroplast genome can express >120 foreign genes from different organisms, including bacteria, viruses, fungi, animals, and humans. The insertion of commercially useful traits, including herbicide and insect resistance, into soybean resulted in high-level expression and superior transgene containment, with no antibiotic selectable markers; but even so, these lines were not developed commercially. Nevertheless, recurring concerns about insect resistance against biopesticides have resulted in new USDA requirements on planting *Bt* corn [122], which may eventually require utilization of the transplastomic approach to confer agronomic traits. The nuclear transgenic approach is inadequate to develop products when higher-level transgene expression is a requirement. Thus, chloroplast transformation has a unique advantage in advancing the field of molecular farming for the production of vaccines, biopharmaceuticals, or other bio-products.

Although products with high-level expression have now advanced to the clinic or are in commercial development, a better understanding of chloroplast translation is required to improve several other gene products. The availability of chloroplast genome sequences should help in the development of codon optimization programs using highly expressed chloroplast genes, but among the ~3000 cultivated crops, sequenced chloroplast genomes are available for crops from fewer than 70 genera. Major funding agencies have not supported crop chloroplast genome sequence projects because of the misconception that all chloroplast genomes are similar, as evidenced by the publication of fewer than ten crop chloroplast genome sequences between 1986 and 2004. This review illustrates the importance of sequencing more crop chloroplast genomes for various biotechnology applications. Furthermore, new selectable markers are needed to transform the chloroplast genomes of cereals, which has been elusive for the past two decades.

Chloroplast genome sequences will be valuable assets in herbal medicine. Most medicinal plants are rare species and very little information is available to confirm their identity. DNA barcodes derived from chloroplast genomes will be useful for identifying varieties and resources; this concept is also valuable in the identification of the origin of cultivated crops and their close relatives to enhance breeding or transfer of useful traits. Molecular techniques to sequence the genomes of single chloroplasts could help to eliminate chloroplast-like sequences that are present in the mitochondrial or nuclear genome. The ability to sequence chloroplast genomes using minimal leaf materials could help us to understand variations in different segments of a variegated leaf in horticultural crops. Further, determining complete chloroplast genome sequences from fossils or recently extinct plants could shed more light on chloroplast genome evolution; help us to understand these species’ inadequate fitness to cope with environmental changes; and help us to build new phylogenetic trees. The technology for isolating DNA from fossils is already available [166–168]. All of these goals can be accomplished with less expensive and more accurate genome sequences, utilizing longer read sequencing technology and new bioinformatics tools.

## Abbreviations

ACE2, Angiotensin-converting enzyme 2; BAC, bacterial artificial chromosome; *Bt*, *Bacillus thuringiensis*; CTB, Cholera toxin B subunit; cGMP, Current Good Manufacturing Processes; CTB, Cholera toxin B subunit; dsRNA, double-stranded RNA; FIX, clotting factor IX; *infA*, *translation initiation factor 1*; IR, inverted repeat; LSC, large single copy; ndh, NAD(P)H dehydrogenase; NCBI, National Center for Biotechnology Information; NGS, next-generation sequencing; RNAi, RNA interference; SSC, small single copy; γ-TMT, γ-Tocopherol methyltransferase; TSP, total soluble protein; UTR, untranslated region; Ang (1–7), Angiotensin (1–7)

## Additional file

Additional file 1: Table S1. The chloroplast genes which are absent in specific species, their knock out phenotypes and transfer to nuclear genomes. (DOCX 23 kb)
