# Supplementary material for: Chloroplast genomes: diversity, evolution, and applications in genetic engineering
Source: Genome Biol. 2016 Jun 23;17:134. doi: 10.1186/s13059-016-1004-2 (PMC4918201; doi:10.1186/s13059-016-1004-2)
Supplement: Additional file 1: Table S1. — The chloroplast genes which are absent in specific species, their knock out phenotypes and transfer to nuclear genomes. (DOCX 23 kb) [file 13059_2016_1004_MOESM1_ESM.docx]

| **Gene** | **Function** | **Chloroplast genome  reference** | **Phenotype of tobacco  knock out transformants** | **Tobacco knock out  transformants reference** | **Transfer to**  **nuclear genome** | **Transfer  reference** |
| --- | --- | --- | --- | --- | --- | --- |
| *accD* | Acetyl-CoA carboxylase beta subunit | [1] | Essential | [11] | Yes/Trifolium | [1] |
| *infA* | Chloroplast translation initiation factor | [2] | ND | ND | Yes/Arabidopsis | [2] |
| *psaI* | photosystem I subunit I | [1] | ND | ND | No/Lathyrus | [1] |
| *psbJ* | photosystem II subunit J | NC_027076 | bleached in high light condition | [12] | ND | ND |
| *rpl18* | ribosomal protein L18 | [3] | Essential | [13] | ND | ND |
| *rpl20* | ribosomal protein L20 | [3] | Essential | [14] | ND | ND |
| *rpl22* | ribosomal protein L22 | [4] | Essential | [15] | Yes/Rosid | [4] |
| *rpl23* | ribosomal protein L23 | [5] | Essential | [15] | Yes/spinach | [20] |
| *rpl32* | ribosomal protein L32 | [6] | Essential | [15] | Yes/Thalictrum coreanum | [6] |
| *rpl33* | ribosomal protein L33 | [7] | cold sensitive | [14] | No/Vigna radiata | NCBI database |
| *rpoA* | RNA polymerase alpha subunit | [3] | albino | [16] | Yes/Physcomitrella | [21] |
| *rps14* | ribosomal protein S14 | [8] | Essential | [17] | ND | ND |
| *rps16* | ribosomal protein S16 | [9] | Essential | [15] | Yes/Medicago | [9] |
| *rps19* | ribosomal protein S19 | [10] | ND | ND | ND | ND |
| *rps2* | ribosomal protein S2 | NC_020321 | Essential | [14] | ND | ND |
| *ycf1* | unknown | [3] | Essential | [18] | No/rice | NCBI database |
| *ycf2* | unknown | [3] | Essential | [18] | No/rice | NCBI database |
| *ycf4* | photosystem I assembly protein | [1] | growth retard | [19] | No/Lathyrus | [1] |
| ND: No data | | | | | | |

**Additional file 1: Table S1: The chloroplast genes which are absent in specific species, their knock out phenotypes and transfer to nuclear genomes.**

**References**

1. Magee AM, Aspinall S, Rice DW, Cusack BP, Sémon M, Perry AS, et al. Localized hypermutation and associated gene losses in legume chloroplast genomes. Genome Res. 2010; 20:1700–10.
2. Millen RS, Olmstead RG, Adams KL, Palmer JD, Lao NT, Heggie L, et al. Many parallel losses of infA from chloroplast DNA during angiosperm evolution with multiple independent transfers to the nucleus. Plant Cell. 2001; 13:645–58.
3. Jansen RK, Cai Z, Raubeson LA, Daniell H, Leebens-Mack J, Müller KF, et al. Analysis of 81 genes from 64 plastid genomes resolves relationships in angiosperms and identifies genome-scale evolutionary patterns. Proc Natl Acad Sci U S A. 2007; 104:19369–74.
4. Jansen RK, Saski C, Lee SB, Hansen AK, Daniell H. Complete plastid genome sequences of three rosids (*Castanea*, *Prunus*, *Theobroma*): evidence for at least two independent transfers of rpl22 to the nucleus. Mol Biol Evol. 2011; 28:835–47
5. Lin CP, Wu CS, Huang YY, Chaw SM. The complete chloroplast genome of *Ginkgo biloba r*eveals the mechanism of inverted repeat contraction. Genome Biol Evol. 2012; 4:374–81.
6. Park S, Jansen RK, Park S. Complete plastome sequence of *Thalictrum coreanum* (Ranunculaceae) and transfer of the rpl32 gene to the nucleus in the ancestor of the subfamily Thalictroideae. BMC Plant Biol. 2015; 15:40.
7. Guo X, Castillo-Ramírez S, González V, Bustos P, Fernández-Vázquez JL, Santamaría RI, et al. Rapid evolutionary change of common bean (*Phaseolus vulgaris* L) plastome, and the genomic diversification of legume chloroplasts. BMC Genomics. 2007; 8:228.
8. Cahoon AB, Sharpe RM, Mysayphonh C, Thompson EJ, Ward AD, Lin A. The complete chloroplast genome of tall fescue (*Lolium arundinaceum*; Poaceae) and comparison of whole plastomes from the family Poaceae. Am J Bot. 2010; 97:49–58.
9. Ueda M, Nishikawa T, Fujimoto M, Takanashi H, Arimura S, Tsutsumi N, et al. Substitution of the gene for chloroplast RPS16 was assisted by generation of a dual targeting signal. Mol Biol Evol. 2008; 25:1566–75.
10. Middleton CP, Senerchia N, Stein N, Akhunov ED, Keller B, Wicker T, et al. Sequencing of chloroplast genomes from wheat, barley, rye and their relatives provides a detailed insight into the evolution of the Triticeae tribe. PLoS One. 2014; 9:e85761.
11. Kode V, Mudd EA, Iamtham S, Day A. The tobacco plastid accD gene is essential and is required for leaf development. Plant J. 2005; 44:237–44.
12. Swiatek M, Regel RE, Meurer J, Wanner G, Pakrasi HB, Ohad I, Herrmann RG. Effects of selective inactivation of individual genes for low-molecular-mass subunits on the assembly of photosystem II, as revealed by chloroplast transformation: the psbEFLJ operon in *Nicotiana tabacum*. Mol Genet Genomics. 2003; 268:699–710.
13. Bryant N, Lloyd J, Sweeney C, Myouga F, Meinke D. Identification of nuclear genes encoding chloroplast-localized proteins required for embryo development in Arabidopsis. Plant Physiol. 2011; 155:1678–89.
14. Rogalski M, Ruf S, Bock R. Tobacco plastid ribosomal protein S18 is essential for cell survival. Nucleic Acids Res. 2006;34:4537–45.
15. Fleischmann TT, Scharff LB, Alkatib S, Hasdorf S, Schöttler MA, Bock R. Nonessential plastid-encoded ribosomal proteins in tobacco: a developmental role for plastid translation and implications for reductive genome evolution. Plant Cell. 2011; 23:3137–55.
16. De Santis-MacIossek G, Kofer W, Bock A, Schoch S, Maier RM, Wanner G, et al. Targeted disruption of the plastid RNA polymerase genes rpoA, B and C1: molecular biology, biochemistry and ultrastructure. Plant J. 1999; 18:477–89.
17. Ahlert D, Ruf S, Bock R. Plastid protein synthesis is required for plant development in tobacco. Proc Natl Acad Sci U S A. 2003;100:15730–5.
18. Drescher A, Ruf S, Calsa T Jr, Carrer H, Bock. R. The two largest chloroplast genome-encoded open reading frames of higher plants are essential genes. Plant J. 2000; 22:97–104.
19. Krech K, Ruf S, Masduki FF, Thiele W, Bednarczyk D, Albus CA, et al. The plastid genome-encoded Ycf4 protein functions as a nonessential assembly factor for photosystem I in higher plants. Plant Physiol. 2012; 159: 579–91.
20. Bubunenko MG, Schmidt J, Subramanian AR. Protein substitution in chloroplast ribosome evolution: A eukaryotic cytosolic protein has replaced its organelle homologue (L23) in spinach. J Mol Biol. 1994; 240: 28–41.
21. Sugiura C, Kobayashi Y, Aoki S, Sugita C, Sugita M. Complete chloroplast DNA sequence of the moss *Physcomitrella patens*: evidence for the loss and relocation of rpoA from the chloroplast to the nucleus. Nucleic Acids Res. 2003; 31: 5324–31.
